# Low-volume versus high-volume initiated trans-anal irrigation therapy in adults with chronic constipation: study protocol for a randomised controlled trial

**DOI:** 10.1186/s13063-017-1882-y

**Published:** 2017-03-31

**Authors:** Christopher Emmett, Helen Close, James Mason, Shiva Taheri, Natasha Stevens, Sandra Eldridge, Christine Norton, Charles Knowles, Yan Yiannakou

**Affiliations:** 1 University Hospital of North Durham, North Road, Durham, DH1 5TW UK; 2 School of Medicine, Pharmacy and Health, Durham University Queen’s Campus, University Boulevard, Thornaby, Stockton-on-Tees, TS17 6BH UK; 3 WMS – Population Evidence and Technologies, University of Warwick, Coventry, CV4 7AL UK; 4 Blizard Institute, Queen Mary University of London, 1st Floor Abernethy Building, 2 Newark St, London, E1 2AT UK; 5 Blizard Institute, Barts and The London School of Medicine and Dentistry, Yvonne Carter Building, 58 Turner St., London, E1 2AB UK; 6 Kings College London, 2.25 James Clerk Maxwell Building, 57 Waterloo Road, London, SE1 8WA UK

**Keywords:** Constipation, Irrigation, Chronic

## Abstract

**Background:**

Constipation is common in adults and up to 20% of the population report this symptom. Chronic constipation (CC), usually defined as more than 6 months of symptoms, is less common but results in 0.5 million UK GP consultations per annum. The effect of symptoms on measured quality of life (QOL) is significant, and CC consumes significant health care resources. In the UK, it is estimated that 10% of district nursing time is spent on constipation. Trans-anal irrigation therapy has become a widely used treatment despite a lack of robust efficacy data to support its use. The long-term outcome of treatment is also unclear. A randomised comparison of two different methods of irrigation (high- and low-volume) will provide valuable evidence of superiority of one system over the other, as well as providing efficacy data for the treatment as a whole.

**Methods:**

Participants will be recruited based on predetermined eligibility criteria. Following informed consent, they will be randomised to either high-volume (HV) or low-volume (LV) irrigation and undergo standardised radiological and physiological investigations. Following training, they will commence home irrigation with the allocated device. Data will be collected at 1, 3, 6 and 12 months according to a standardised outcomes framework. The primary outcome is PAC-QOL, measured at 3 months. The study is powered to detect a 10% difference in outcome between systems at 3 months; this means that 300 patients will need to be recruited.

**Discussion:**

This study will be the first randomised comparison of two different methods of trans-anal irrigation. It will also be the largest prospective study of CC patients treated with irrigation. It will provide evidence for the effectiveness of irrigation in the treatment of CC, as well as the comparative effectiveness of the two methods. This will enable more cost-effective and evidence-based use of irrigation. Also, the results will be combined with the other studies in the CapaCiTY programme to generate an evidence-based treatment algorithm for CC in adults.

**Trial registration:**

ISRCTN, identifier: ISRCTN11093872. Registered on 11 November 2015. Trial not retrospectively registered. Protocol version 3 (22 January 2016).

**Electronic supplementary material:**

The online version of this article (doi:10.1186/s13063-017-1882-y) contains supplementary material, which is available to authorized users.

## Background

### Burden of disease

Constipation is common in adults and up to 20% of the population report this symptom depending on the definitions used [1–3], with a higher prevalence in women [1, 4, 5] and older people [6, 7]. Chronic constipation (CC), usually defined as more than 6 months of symptoms, is less common [8] but results in 0.5 million UK GP consultations per annum. A proportion of the population suffer symptoms which are both chronic and more disabling (about 1–2% of the population) [9]. Such patients, who are predominantly female [10], are usually referred to secondary care with many progressing to tertiary specialist investigation. Patient dissatisfaction is high in this group; nearly 80% feel that laxative therapy is unsatisfactory [11] and the effect of symptoms on measured quality of life (QOL) is significant [12]. CC consumes significant health care resources. In the US in 2012, a primary complaint of constipation was responsible for 3.2 million physician visits [13] resulting in (direct and indirect) costs of US$1.7 billion. In the UK, it is estimated that 10% of district nursing time is spent on constipation [14] and the annual spend on laxatives exceeds £80 million, with 17.4 million prescriptions being issued in 2012 (Health and Social Care Information Centre, 2013) [15].

### Pathophysiological basis of chronic constipation

The act of defaecation is dependent on the coordinated functions of the colon, rectum and anus. Considering the complexity of neuromuscular (sensory and motor) functions required to achieve planned, conscious and effective defaecation [16] it is no surprise that disturbances to perceived ‘normal’ function occur commonly at all stages of life. Clinically, such problems commonly lead to symptoms of obstructed defaecation, e.g. straining; incomplete, unsuccessful or painful evacuation; bowel infrequency; abdominal pain and bloating. After exclusion of a multitude of secondary causes (obstructing colonic lesions, neurological, metabolic and endocrine disorders), the pathophysiology of CC can broadly be divided into problems of colonic contractile activity and thus stool transit and problems of the pelvic floor. Thus, with specialist physiological testing (using a standard panel of radio-physiological tests of colonic and anorectal function, hereafter referred to as INVEST in this protocol), patients may be divided into those who have slow colonic transit, evacuation disorder, both or neither (no abnormality found with current tests). Evacuation disorders can be then subdivided into those in which a structurally significant pelvic floor abnormality is evident, e.g. rectocoele or internal prolapse (intussusception) and those in which there is a dynamic failure of evacuation without structural abnormality: most commonly termed ‘functional defaecation disorder (FDD)’.

### Chronic constipation management overview

Management of CC is a major problem due to its high prevalence and lack of widespread specialist expertise. In general, a step-wise approach is undertaken, with first-line conservative treatment, such as lifestyle advice and laxatives (primary care), followed by nurse-led bowel retraining programmes, sometimes including focussed biofeedback and psychosocial support (secondary/tertiary care). Although these treatments may improve symptoms in more than half of patients, they are very poorly standardised in the UK and are not universally successful [17]. Thus, patients with intractable symptoms and impaired QOL may be offered a range of costly, irreversible surgical interventions with unpredictable results [18, 19], sometimes resulting in major adverse events (AEs) or a permanent stoma.

### Overall rationale for the CapaCiTY programme

The current trial forms part of an NIHR-funded programme (PGfAR: RP-PG-0612-20001). This programme aims to develop the evidence base for the management of CC in adults which is currently lacking. This is in contrast to the management of CC in children for which NICE guidance has been recently published (http://pathways.nice.org.uk/pathways/constipation/clinical-management-of-idiopathic-constipation-in-children-and-young-people) [20, 21]; and for adults with faecal incontinence (http://pathways.nice.org.uk/pathways/faecal-incontinence). Thus, the current situation is one where there are considerable variations in practice, particularly in specialist services. With a number of new drugs gaining or seeking NHS approval [22–25] and technologies at a horizon-scanning stage [18, 26, 27] it is timely that the currently limited evidence base for adult CC is developed for resource-constrained NHS providers to have confidence that new and sometimes expensive investigations and therapies are appropriate and cost-effective. A cost-conscious pathway of care may help to reduce health care expenditure by appropriately sequencing the care provided, while targeting more expensive therapies at those most likely to benefit. Such data will inform the development and commissioning of integrated care pathways. An overview of the CapaCiTY programme is provided as a scheme (See Additional file 1) and includes a series of interlinked work package signature pages (WPs) that answer the important questions for patient care. A rolling programme of national recruitment will provide a large cohort of well-defined patients for subsequent studies within sequential WPs over 5 years. The focus will be on generating real-life evidence from pragmatic studies which will provide valid clinical outcome measures, patient acceptability and cost. Armed with such data it will be possible to develop an NHS management algorithm for CC which will meet patient, clinician and policy aims.

### Specific clinical background to the prospective cohort study of trans-anal irrigation (TAI)

Anal irrigation, using a variety of commercially available devices, has been rapidly disseminated internationally over the past 3–5 years, first in patients with neurological injury [28, 29] and subsequently in other CC groups [30, 31]. Despite a lack of published data other than from small selected case series, it is now available on the drug tariff and generally considered to be the next step in patients failing other nurse-led interventions such as biofeedback. Anal irrigation has permeated the UK market without robust efficacy data and with on-going concerns regarding longevity of treatment and complications [28, 32]. Retrospective clinical audit data and review [32] suggest a continued response rate after 1 year of approximately 50% with such patients, thus avoiding or delaying surgical intervention. An accurate assessment of response rate and acceptability of this intervention requires confirmation in a large prospective cohort, together with clinico-physiological predictors of success. In addition, two alternative systems for delivery of TAI exist; low-volume systems delivering approximately 70 ml per irrigation, and high-volume systems delivering up to 2 L of irrigation (although typically only 0.5–1.5 L is required per irrigation). The low-volume system is cheaper, costing approximately £750 p.a. based on alternate-day use, compared with approximately £1400–1900 for high-volume irrigation, and may be more acceptable to patients, and so a randomised study comparing the two systems is needed.

### Trial design: rationale

Robust data for the use of TAI therapy in chronic (idiopathic) constipation are lacking. In addition, there are no data demonstrating superiority of high-volume irrigation over low-volume systems. Given the differences in cost between the two systems, a randomised study of well-characterised patients comparing the two methods would provide useful information on whether one system holds a clear advantage over the other. Also, the short- and long-term efficacy and acceptability of therapy in CC could be evaluated. This is timely and informative given the rapidly increasing popularity of this treatment and the fact that TAI is an invasive therapy for which patient selection should also be optimised to maximise benefit.

In practice, patients will use one system only (plus defined ‘rescue therapies’ – see below) for a minimum of 3 months. After this time point they may switch to the other system if their initial therapy was ineffective/unsatisfactory. Thus, consenting patients will be randomised to initiate therapy with one of these systems but will have the option of switching to the other after an initial 3-month period. This allows us to identify response rates to each system in the short term (3 months), and thereafter this study is a comparison between treatment strategies (low-volume initiated therapy versus high-volume initiated therapy) rather than a pure comparison of the two techniques. This is a patient-centred study design aiming to limit the time that patients spend using ineffective therapy without being allowed to try an alternative. This also allows estimation of comparative cost-effectiveness of the two treatment pathways, and whether one system works better depending on the radio-physiological profile of the patient. Recent data estimates that approximately 85% of patients are still using irrigation at 1 month; this represents a significant short-term treatment failure rate [33]. Once patients have switched therapy, they may not switch back to the first system; once they have tried both systems and discontinued them then they will be considered to have completed the intervention and they will return to routine clinical care.

Irrigation is a maintenance therapy rather than a cure. In addition to outcome measures of the Patient Assessment of Constipation Quality of Life questionnaire (PAC-QOL) [34, 35] score at 3 months, patients will provide survival data (time until cessation of irrigation therapy due to lack of benefit). Switching systems does not affect this; the survival data is based on the use of irrigation irrespective of system. A survival analysis is appropriate since anal irrigation is time-consuming and inconvenient as a therapy and patients may find the process distasteful. Patients are unlikely to continue with treatment if they are not gaining worthwhile benefit from it; treatment continuation is a useful patient-centric assessment.

Consideration of the findings from both groups (individually and together) will be used to model the net value to patients of anal irrigation, considering persistence of benefit.

### Risks/benefits

The interventions proposed are those already offered to patients in specialist centres throughout the UK and internationally. All interventions pose acceptable and minimal risks. For instance, the only invasive tests (INVEST) have been performed daily in most specialist centres for up to 30 years without any recorded complication (Barts Health experience of over 10,000 patients). A small ionising radiation dose is required for one of the tests (covered below). A number of questionnaires contain personal questions about bowel problems and the effect of these on QOL and psycho-behavioural functioning; however, all have been used in studies of similar patients previously.

Trans-anal irrigation has been shown to be a low-risk intervention and is widely used in a variety of defaecatory disorders such as neurogenic bowel dysfunction, idiopathic constipation and faecal incontinence. Serious adverse events (SAEs) are rare, with one study reporting two nonfatal bowel perforations out of approximately 110,000 irrigation treatments [28]. Other potential side effects include pain, bleeding, painful haemorrhoids and anal fissure. A recent study reported an overall adverse event (AE) rate of 22% when all minor and reversible events were considered. Thirteen percent reported technical problems with equipment and 13% reported minor side effects/AEs [33]_._ The risk of nonparticipation is considered very low.

The benefits of participation are that patients will receive a very high standard of monitored care as a consequence of the detailed protocol. Participation will inform future treatment options for patients with CC.

## Trial objectives

### Primary objectives

1. To compare the impact upon patient disease-specific QOL of TAI initiated with a low-volume versus a high-volume system in patients with CC, measured at 3 months

### Secondary objectives

To determine:

1. Survival (continuation of benefit) and acceptability in the longer term (up to 12 months)
2. Disease-specific outcomes at 1, 3, 6 and 12 months
3. The influence of patient characteristics (urge to defaecate, balloon sensory testing results) upon treatment success, and response by type of system used
4. The acceptability of each system to patients
5. Strategies for tailoring therapy to meet patients’ individual needs, and the factors involved in this
6. The safety of each system and prospective tracking of AEs
7. The cost-effectiveness of care
8. Qualitatively evaluation of patient and health professional experience

## Methods

### Setting

Specialist centres across the UK with a mix of urban and rural referral bases

### Recruiting sites (initial)

- Barts Health NHS Trust [Allison]
- St. Mark’s Hospital at London North West Healthcare NHS Trust [Vaizey]
- University College Hospital London [Emmanuel]
- Guys and Thomas’ Hospitals London [Williams]
- Sandwell and West Birmingham NHS Trust [Gill]
- County Durham and Darlington NHS Foundation Trust [Yiannakou]
- University Hospital Southampton NHS Foundation Trust [Nugent]
- Norfolk and Norwich University Hospitals NHS Foundation Trust [Speakman]
- University Hospital of South Manchester NHS Foundation Trust [Telford]
- Sheffield Teaching Hospital NHS Foundation Trust [Brown]
- North Bristol NHS Foundation Trust [Dixon]
- University Hospitals Bristol, NHS Foundation Trust [Mabey/Randall]
- Newcastle Upon Tyne, NHS Foundation Trust [Plusa]
- Homerton University Hospital, NHS Foundation Trust [Cuming]

### Reserve sites

- University Hospital Leicester NHS Foundation Trust [Miller]

### Central facilities

- Bart’s and the London, Pragmatic Clinical Trials Unit. Centre for Primary Care and Public Health, Queen Mary University London (QMUL)
- County Durham and Darlington NHS Foundation Trust, Durham Clinical Trials Unit. Wolfson Research Institute, Durham University

### Inclusion criteria

- Age 18–70 years
- Patient self-reports problematic constipation
- Symptom onset more than 6 months before recruitment
- Symptoms meet American College of Gastroenterology definition of constipation
- Nonresponse to constipation treatment to a minimum basic standard (see NHS Map of Medicine 2012) [36]: Comprising lifestyle *and* dietary measures *and* two or more laxatives or prokinetics tried (no time requirement)
- Ability to understand written and spoken English (due to questionnaire validity)
- Ability and willingness to give informed consent
- Failure of previous nurse-led behavioural therapy
- Ability of patient/carer to use anal irrigation

The study will use the American College of Gastroenterology definition of constipation [37] (which is reasonable, simple and extensively published): unsatisfactory defaecation characterised by infrequent stool, difficult stool passage or both for at least previous 3 months. This avoids the more complex Rome definitions.

### Exclusion criteria

The study interventions necessitate the exclusion of major causes of secondary constipation. In detail:

- Significant organic colonic disease (‘red flag’ symptoms, e.g. rectal bleeding previously investigated); inflammatory bowel disease (IBD); megacolon or megarectum (if diagnosed beforehand) (the study will provide a useful estimate of the prevalence of such cases in referral practice); severe diverticulosis/stricture/birth defects deemed to contribute to symptoms (incidental diverticulosis not an exclusion)
- Major colorectal resectional surgery
- Current overt pelvic organ prolapse (bladder, uterus, vagina, rectum) or disease requiring surgical intervention
- Previous pelvic floor surgery to address defaecatory problems: posterior vaginal repair, STARR and rectopexy; previous sacral nerve stimulation
- Previous use of TAI therapy to treat constipation
- Rectal impaction (as defined by digital and abdominal examination: these form part of the NHS Map of Medicine basic standard) [36]
- Significant neurological disease deemed to be causative of constipation, e.g. Parkinson’s disease, spinal injury, multiple sclerosis, diabetic neuropathy (not uncomplicated diabetes alone)
- Significant connective tissue disease: scleroderma, systemic sclerosis and SLE (not hypermobility alone)
- Significant medical comorbidities and activity of daily living impairment (based on Bartell index in apparently frail patients [38], Barthel Index ≤11)
- Physical disability/impairment which prevents the use of one or other of the irrigation devices
- Major psychiatric diagnosis (schizophrenia, major depressive illness, mania, self-harm, drug/alcohol addiction)
- Chronic regular opioid use (at least once daily use) where this is deemed to be the cause of constipation based on temporal association of symptoms with onset of therapy; all regular strong opioid use
- Pregnancy or intention to become pregnant during study period

Note: ‘red flag’ symptoms are not an exclusion if they have been investigated before enrollment and organic disease excluded. Previous TAI therapy does not include private (non-NHS) ‘colonic irrigation’ therapy; prior use of such treatments is not an exclusion criterion.

### Study interventions: trans-anal irrigation therapy

Trans-anal irrigation training will be provided by trained nurse or physiotherapist with experience in delivering care for CC. They must have initiated irrigation therapy in at least three patients independently, and be a nurse/therapist of good standing within a clinical team regularly seeing patients with CC. A standardised approach and intervention will be provided via the use of an intervention manual. For the first 3 months of participation in the study, patients may not use other therapies besides anal irrigation and those rescue therapies specified below. They may discontinue therapy at any point (elective withdrawal from intervention) and may switch from one system to the other after 3 months. Switching anal irrigation systems before completing the 3-month waiting period will be discouraged. If it does occur, it will be documented as a protocol deviation with the timing and reason documented. If symptoms are severe despite the use of irrigation and rescue therapies then other medications may be used on compassionate grounds, but this must be recorded in the Case Report Form(CRF)/concomitant medications log.

The course of therapy will include a nurse-led training session (or more if required to ensure that the device is being used effectively) followed by patient-led home irrigation therapy. The low-volume system commonly used in practice is Qufora® Mini (MBH-International). Various high-volume systems are used, all of which have very similar mechanisms of action; these include Peristeen™ (Coloplast) and Qufora Toilet/Qufora Balloon™ (MBH-international). These are commercially available TAI systems available on prescription in NHS practice.

#### Low-volume irrigation

This system consists of a small reservoir attached to a cone. The reservoir holds approximately 70 ml of water and is squeezed to inject water into the rectum. The regime used will be as follows: initial irrigation once daily for 14 days using one to three insufflations (each of 70 ml approximately). This may then be reduced to alternate days depending on response. Patients may then adjust frequency and volume depending on response. They may irrigate as much and as often as they feel is necessary to give them benefit and this information will be captured on the CRF with the aid of an Irrigation Journal.

#### High-volume irrigation

High-volume systems consist of an irrigation bag connected to a tube. The water flows into the rectum, either by gravity or using a pump. Some systems employ a balloon to hold the device in place during irrigation; others require the patient to hold it in place. The mechanism of action is the same for all systems. Initial frequency of irrigation is the same as for low-volume irrigation; i.e. daily for 14 days, then alternate days. Patients will commence with irrigations of 300 ml and increase this by 100 ml every 2 days until satisfactory defaecation is achieved or the procedure becomes uncomfortable, up to a maximum of 1500 ml. Patients may adjust therapy depending on response, as for low-volume irrigation.

#### Switching between anal irrigation systems

After 3 months of using one system, patients may switch to the other or discontinue therapy and return to routine clinical care. This will be entirely patient-led, and reasons for changing systems will be explored during follow-up visits and captured on the CRF. There is, therefore, no defined protocol for switching treatments as patients may do this for any reason; analysis of time to switching/discontinuing therapy, as well as the patient-reported reasons for doing so, will provide insight into why each irrigation system is or is not successful. In addition, qualitative interviews with patients who have switched or discontinued therapy will be used to explore these issues more deeply.

### Endpoints

#### Clinical endpoints

All clinical endpoints will be in common with a single standardised outcome framework (consistently used within all CapaCiTY programme studies). All outcomes will be recorded at baseline, 3, 6 and 12 months in face-to-face clinics (or by telephone call if necessary). PAC-QOL, the Patient Assessment of Constipation Symptoms (PAC-SYM) and the EuroQol Health Outcome measure (EQ-5D-5 L) and the EuroQol Visual Analogue Scale (EQ-VAS) will additionally be collected at 1 month; this is to capture reasons for early nonresponse to therapy, as well as to better characterise the patient group and provide more data for economic analysis. The primary endpoint will be at 3 months.

### Primary clinical outcome

- Patient Assessment of Constipation Quality of Life questionnaire (PAC-QOL [34, 35]) at 3 months.

### Secondary clinical outcomes

- PAC-QOL score and individual domain scores at 1, 3, 6 and 12 months
- Time to cessation of each system of irrigation; total time in treatment with either system (from Irrigation Journal) at 1, 3, 6 or 12 months
- Reason for cessation (of each system) (Irrigation Journal and qualitative interviews) at 1, 3, 6 and 12 months
- Patient Assessment of Constipation Symptoms (PAC-SYM): aggregate and domain scores at 1, 3, 6 and 12 months
- Volume and duration of irrigation (Irrigation Journal) at 1, 3, 6 and 12 months
- Number and nature of bowel motions (captured in 2-week Patient Diary) at 3, 6 and 12 months
- Symptom scores derived from Diary records (taken over 2 weeks before or around each follow-up contact. These will include number of spontaneous complete bowel motions at 3, 6 and 12 months
- Generalised Anxiety Disorder Questionnaire (GAD-7) at 3, 6 and 12 months
- Depression, anxiety and somatisation modules of the PHQ-9 at 3, 6 and 12 months
- Global patient satisfaction/improvement score (Visual Analogue Scale; VAS) at 3, 6 and 12 months
- Patient acceptability and recommendation to other patients (qualitative interviews)
- Behavioural Response to Illness Questionnaire (CC-BRQ) and Brief Illness Perception Questionnaire BIPQ (CC) at 3, 6 and 12 months
- Generic quality of life: EuroQol EQ-5D-5 L and EQ-VAS scores 1, 3, 6, and 12 months
- Use of health care resources, AEs, and concomitant medications (collected using Patient Journal) at 3, 6 and 12 months

#### Health economic outcomes

- Interventions, treatment sequelae and other health resource use related to the care of CC will be recorded in natural units and cost applied where possible using national reference costs. Additionally, patient costs related to constipation and the opportunity cost of time away from normal activities will be valued using national reference sources.

#### Patient experience (see ‘Qualitative interviews’)

- Face-to-face, digitally recorded, semistructured interviews will be conducted involving a purposive, diverse sample of patients throughout the programme, with participants reflecting a range of ages, geographical locations and, where possible, other pertinent attributes, such as ethnicity and gender, continuing until data saturation when no new themes emerge. Participants will be approached by a member of the research team and will undergo a separate consent process if they are willing to participate in the qualitative study.

### Study design/plan – Study visits

**Fig. 1:**
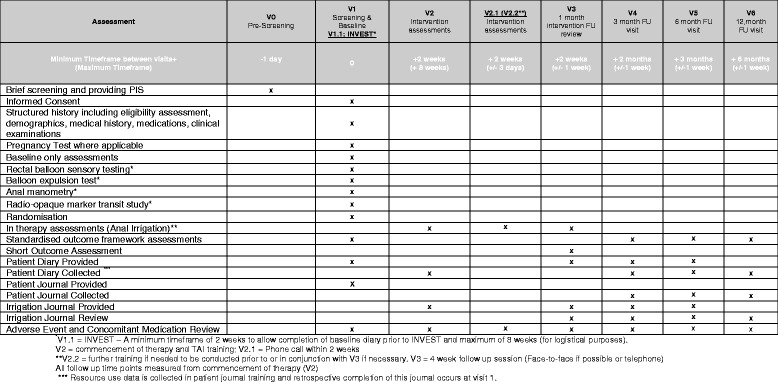
SPIRIT figure (schedule of assessments)

The following section provides an overview of patient study visits. This is provided in diagrammatic format in the attached Standard Protocol Items: Recommendations for Interventional Trials (SPIRIT) figure (Fig. 1. See Additional file 2 for the SPIRIT Checklist).

### Visit 0: Prescreening: eligibility assessment

A Good Clinical Practice (GCP)-trained and delegated local researcher will screen for basic eligibility by telephone (or face-to-face interview based on patient choice). Potentially eligible patients will be identified either in clinic, from referral letters from GPs/other consultants to the constipation clinic, and from patients participating in CapaCiTY 01 who did not respond, or have ceased to respond, to habit training (HT)/biofeedback (HTBF). Participants will be provided with adequate explanation of the aims, methods, anticipated benefits and risks of anal irrigation therapy and will take away or be posted an invitation letter and a Participant Information Sheet (PIS). Patients will be given at least 24 h to consider participation and invited to attend clinic for visit 1 (see below).

The study screening number will be allocated as follows:

- Study code 02
- Site code – three-letter code for each site
- Participant Code – four-digit code given consecutively and attributed at each site

For example, the first participant recruited at Barts Health Trust would be assigned the code 02-BLT-0001. Patients progressing to other studies within the CapaCiTY programme will keep this number for pathway tracking.

### Visit 1: Screening, consent, baseline assessments and randomisation

Visit 1 will be conducted face to face in clinic. Following a detailed discussion about the trial, potentially eligible and agreeable patients will complete a written informed consent, followed by a more thorough screening and confirmation of eligibility for randomisation by standardised medical and surgical history and physical examination (the latter if not already performed within the previous 3 months).

Patients who decide not to opt for treatment will be invited to offer reasons and these will be recorded when provided. Patients declining participation will continue to receive usual care as locally provided. There is no obligation for patients to give reasons for nonparticipation.

For those patients entering the study, additional baseline outcome assessments will be conducted. These include several key validated assessments that profile patient characteristics, informing disease pathophysiology and potential predictors of treatment response. All have been selected on the basis of trade-off between adequate detail and achievable brevity. These instruments will be combined with the standardised outcome framework into a single booklet (design and presentation have been optimised by patient representatives).

#### Confirmation of eligibility

- Standardised history by interview including previous medication usage
- Clinical examination findings (carried forward if performed previously within last 3 months): standardised exam of perineum/anus/rectum

#### Baseline outcome assessments

- Baseline outcome assessments (PAC-QOL, PAC-SYM, EQ-5D-5 L and EQ-VAS, PHQ-9, GAD-7, CC-BRQ and BIPQ-CC, see endpoints above)
- Baseline 2-week Patient Diary will be given. Training in completion of the diary will be conducted at visit 1 and the diary will be completed at home and returned at visit 2
- Training and retrospective completion of the Patient Journal will occur at visit 1 for collection of resource data. Prospective completion will occur continuously, with review at each follow-up visit from 3 to 12 months

#### Other baseline only assessments

- Constipation (2006) and IBS (2006) modules of the Rome III Questionnaire
- Cleveland Clinic Constipation Questionnaire
- Brief, chronic pain, autonomic and joint hypermobility assessments
- St. Mark’s Incontinence Score (for concurrent symptoms)

#### Randomisation

Conducted by a member of the research team.

#### INVEST radio-physiology investigations

There is no defined time period for this, but it is suggested that INVEST should be completed within 4 weeks of the visit 1 baseline visit to allow for diary completion before stopping laxatives for INVEST. A maximum of 8 weeks is tolerated to conduct INVEST. Those with INVEST completed in the previous 12 months do not need these repeated and can be booked for visit 2, commencing in a minimum of 2 weeks to allow completion of the baseline diary.

### Training sessions (45–60 min) (V2–V3)

This will use a standardised proforma and will always be face to face. Patients will receive:

### Visit 2: First training session

Visit 2.0

1. Collection of baseline diary completed before stopping laxative (i.e. before INVEST in patients who need this done)
2. Training in TAI: patients will undergo a single, nurse-led training session before starting treatment. The device will be demonstrated to the patient by the nurse specialist and then the patient will practice setting up the device. The trainer will ensure that the patient knows how to use the device correctly before home irrigation is commenced
3. Training in completion of the Irrigation Journal and provision of the Irrigation Journal to be completed weekly. The Irrigation Journal consists of: volume of water introduced, frequency of use, AEs and side effects, e.g. pain, bleeding
4. The trainer and patient will agree a date for delivery of equipment and commencement of home irrigation. Ideally, this should be the same as the first training visit, but this may not be possible due to delay in supplying irrigation equipment. Any delays should be recorded on a deviation log/note to file (CRF 7/8) to allow data analysis to be adjusted accordingly
5. Start date for home irrigation agreed with the patient (this is to allow for any delay in delivery of equipment). Ideally this should be the same day as visit 2, or within 1 week maximum. If any issues or delays have been encountered, a new commencement date is agreed; this should be recorded as a deviation/note to file (CRF 7/8), along with reasons for delay
6. Regulation/standardisation of laxative use: bisacodyl may be used orally as a rescue therapy (up to 20 mg at night), plus glycerine suppositories, one or two, if needed, if no stool for 3 days. In addition, patients may take Movicol up to a maximum dose of two sachets three times per day (TDS) and/or lactulose up to 15 ml twice per day (BD). Prokinetic drugs and any other drug that the *British National Formulary* (BNF) describes as having laxative effect or herbal teas that contain strong purgatives will be discouraged, but if needed (i.e. if symptoms severe) then these are permitted but use must be recorded in the concomitant medications log. There will be no use of enemas.

#### Visit 2.1: First intervention assessment

A telephone call will be made to the patient 14 days (±3 days) after visit 2 to check that everything is proceeding correctly and to resolve any problems. If, due to delay in obtaining equipment, etc., the patient has not started irrigation at this time then the telephone call (and other follow-up visits) should be rescheduled for 14 days later, and the reason for this recorded on CRF 7/8. Adverse events and concomitant medications will also be reviewed.

#### Visit 2.2: Second intervention assessment (if needed)

If there are problems then a further face-to-face training session will be offered, including a review of AEs and concomitant medications. This can occur any time before visit 3 (2 weeks ±1 week from visit 2.1) or in conjunction with visit 3 if not before.

Patients will continue the self-administered therapy using a commercially available device until the end of the study. Patients will be followed up until the end of the data collection phase of the study (variable follow-up 12–24 months depending on date of recruitment) or until they decide to discontinue either the therapy or the trial follow-up. Irrigation will be performed at an agreed frequency initially. Once established on this therapy patients may adjust the frequency and volume of irrigation to suit their particular condition.

Information about treatment will be recorded in the Irrigation Journal. Where a patient switches to the other irrigation device or discontinues treatment (patient choice) the reason for this, as well as the duration of therapy, will be documented. If a patient chooses to switch devices, which they may do at any stage after the 3-month follow-up visit, they will receive training in the other device. They will receive a follow-up by the irrigation nurse as required to resolve any outstanding issues and to check progress. This should be documented on the Irrigation Journal and a note to file, (CRF 8) and change/discontinue, (CRF 12) should be completed. However, they will not be asked to repeat the questionnaires and diaries already completed at 1 and 3 months.

### Visit 3: 1-month follow-up review

1. All patients will receive a further training assessment at 2 weeks (±1 week) after visit 2.1, allowing for any delay as described previously (V3). This visit will be combined with collection of PAC-QOL, PAC-SYM and EQ-5D-5 L and EQ-VAS and should be face to face. The Irrigation Journal will be reviewed at this visit. A telephone call is an acceptable alternative if this is not possible
2. Standardised guidance on how to tailor therapy to each patient depending on initial response will be provided to specialist nurses/therapists. Changes in regimen, as well as system, will be documented on the CRF. As outlined previously, switching between irrigation systems before the 3-month visit is discouraged, and represents a protocol deviation. However, it is recognised that some patients may need to switch systems before 3 months: if this occurs it must be recorded on CRF 12 and on the deviation log. Primary outcome analysis at 3 months will be by intention-to-treat

Telephone support will be available from the therapist between visits (number given, office hours only). The therapist will complete the intervention CRF at every visit or patient contact. For contact with patients after the training period, a note to file (CRF 8) should be completed, and the patient will also make a note of any contact in their Irrigation Journal. In the instance of new psychological issues being determined during consultation, referral for psychological support will be deferred until after completion of irrigation training. The exception to this rule would be where there is clinical concern regarding the patient’s acute mental state requiring more urgent intervention (see ‘Criteria for withdrawal from treatment’). Concerns would be raised by the irrigation nurse team to the research team, and these would be evaluated by the principal investigator (PI) (or a medically trained deputy) and appropriate action taken. Further follow-up visits (V4–V8) will be conducted by the research team. If the patient requires further input from the irrigation nurse this may be arranged as per local practices. Any contact and any changes made or advice given regarding irrigation should be recorded in the Patient Journal and the Irrigation Journal.

### Visits 4–6: Follow-up outcome assessments: visits or telephone consultations

A full, standardised outcome framework and health economic dataset will be recorded at baseline, 3, 6 and 12 months (±1 week) after initiation of intervention at visit 2. To maximise completeness of data collected, follow-up visits will be conducted face-to-face in clinic wherever possible. Where this is not possible, a telephone consultation will be used. The Patient Diary and Journal and Irrigation Journal will be provided for review at each follow-up visit.

Patients deciding to switch to the alternative system will be trained in the new system by the irrigation nurse and this will be recorded on the note to file, CRF 8 and change/discontinue, CRF 12. These patients will not need to complete the questionnaires at 1 month and 3 months if they have already done so.

Within the follow-up period at least three attempts via two different methods (e.g. telephone and letter), will be made by research staff to make contact and collect follow-up data at each time point, after which the time point will be recorded as missing.

### Recruitment and strategies for achieving enrollment

Patients attending specialist centres (outpatient clinics, gastrointestinal (GI) physiology units) for constipation and who have already failed to respond to a minimum basic standard of treatment (see above), as well as nurse-led interventions (biofeedback or habit training), will be eligible for recruitment screening based on criteria. Patients will be recruited from those failing treatment in CapaCiTY 01 but also those patients seen outside the trial who have had nurse-led behavioural therapies without response.

Trial posters will be displayed in primary care, pharmacy and community care settings, directing patients to their nearest research site and contact person, as well as the study website for more information, including the PIS. The same posters may be used to advertise the study via newspapers, trial websites, social media, and patient groups such as Bowel and Cancer Research charity.

Patient and Public Involvement (PPI) consultation with CC patients in secondary care has explored the acceptability of this study design, and we have found that this is likely to be acceptable to patients. The proposed rescue therapy and patient diaries/journals used in the study have been reviewed as part of this process. Care has been taken to ensure that the study design is patient-centred, with flexibility of laxative use incorporated into the protocol, as well as the option to switch treatment after 3 months. This aims to ensure that patient experience of the trial is similar to a nontrial patient in terms of treatment received, within the constraints of a randomised trial.

## Study procedures

### Screening, enrollment

A brief screening questionnaire will be used to determine whether patients meet the inclusion and exclusion criteria (see ‘Eligibility’ above). Screening will be performed by suitably trained study personnel to minimise logistic hurdles, and as determined by geographic availability.

The brief screening questionnaire will also be made available on the study website, with the PIS for patients to self-screen and contact their nearest research site if interested in taking part. All basically eligible participants will then undergo a formal face-to-face consent, screening and enrollment session prior to randomisation.

### Randomisation procedures

Patients will be randomised 1:1 into two groups; those who commence therapy with a low-volume device and those starting with a high-volume device. Patients will be stratified by sex and women by centre. Randomisation will be performed by a GCP-trained member of the research team using a bespoke, secure online system developed by the Pragmatic Clinical Trials Unit (PCTU).

### Blinding

Patients and clinicians are necessarily aware of both INVEST and treatment allocations. The need to collect data on frequency and volume of irrigation, as well as reasons for discontinuing or switching between systems, means that assessor blinding is not possible with respect to these outcomes. Those involved in the development of the statistical analysis plan (SAP) will not have access to any data that will lead them to become unblended and, therefore, they will remain blind. Any researcher collecting CRFs, handling journals or performing statistical analysis on the above outcomes will be unblinded. However, in order to control for observer bias, the primary outcome (PAC-QOL at 3 months) will be concealed; the patients will complete this questionnaire without a researcher present. This will be accomplished in one of the following ways:

1. Direct entry to online secure database, with built-in validation and prompting to ensure data completeness
2. Completing paper questionnaire by following instructions on an information card to ensure that all questions are answered. This will be placed in a sealed envelope marked with the patients pseudonymised study code and will not be opened until the time comes for data entry

### Radio-physiological investigations (INVEST)

Patients will undergo standardised investigations. If INVEST previously conducted within the last 12 months, results can be carried forward. Pregnancy testing will be conducted as per routine NHS practice (10-day NHS rule) in respect to women between menarche and menopause. Women of equivocal status will have a pregnancy test performed as per routine care.

1. Anorectal manometry using standard or high-resolution methods [39–41], depending on local availability, to determine defined abnormalities of recto-anal pressure gradient during simulated evacuation [42–44]
2. Balloon sensory testing using standardised methods [45, 46] (2 ml air per second to maximum 360 ml) to determine volume inflated to first constant sensation, defaecatory desire and maximum tolerated volumes. Rectal hyposensation and hypersensation, defined in accord to gender-specific normative data on 91 healthy adults [47]. The recto-anal inhibitory reflex (RAIR) will also be elicited by a 50-ml rapid inflation (if necessary in 50-ml aliquots up to 150 ml)
3. Fixed volume (50 ml) water-filled rectal balloon expulsion test [42, 43, 48, 49] in the seated position on a commode. Abnormal expulsion is defined as abnormal if failure to expel with a 1-min effort for men and 1.5 min for women [50]
4. Whole gut transit study using serial (different shaped) radio-opaque markers over 3 days with a single plain radiograph at 120 h [51, 52]

Note: INVEST procedures conducted prior to recruitment to the study (i.e. within the past 12 months) may be done using locally available devices and methods.

All patients will undergo TAI therapy irrespective of INVEST results, and will be followed up in the same way. The purpose of INVEST in this study is to identify whether certain radio-physiological results correlate with treatment response, i.e. can we predict likelihood of benefitting from irrigation based on pretreatment investigations. Balloon sensory testing in combination with patient-reported urge to defaecate will be analysed as covariates to determine whether such a relationship is present.

### Concomitant medications

It is inevitable that patients will seek recourse to laxatives and other dietary supplements during the course of the programme. Experience shows that complete prohibition can lead to unreported laxative use, which might confound findings. Although we will strongly discourage ad libitum medication usage and specify a defined breakthrough regimen, we will record cotreatment with sufficient fidelity and integrity to enable use as covariates in analyses using a specific patient journal for this purpose (see ‘Standardised outcome framework’). A concomitant medications list, including a shortlist of contributory or confounding medications, will be used to filtre on data entry. Patients using one system in the medium/long term may wish to revert to the other system or pause treatment for a short period (for example, while going on holiday) for practical reasons. This is permitted but must be recorded in the concomitant medications log. This will not be considered as switching or ending treatments as it is only a short-term measure.

### Criteria for discontinuation

The interventions proposed are well-established in current clinical practice. There are no defined criteria for discontinuation; however, clinicians may withdraw treatment where they have therapeutic or safety concerns, consistent with routine care. Patients may choose to discontinue treatment at any point and return to routine clinical care.

### Procedure for collecting data including Case Report Forms (CRFs) and storage

The data collected for the trial will be a mixture of routinely collected data, verifiable against the medical record and patient-reported outcome (PRO) or questionnaire data, collected directly to CRF.

Each recruiting site will be required to keep accurate and verifiable source notes in the medical record relevant to each study participant’s inclusion and continued participation in the study. Data will be collected, transferred and stored in accordance with GCP guidelines and data protection requirements. The PCTU standard operating procedures (SOPs) and study data management plan will define the exact process of data collection, transfer and storage and control of study data.

A secure online OpenClinica trial database will be provided by the PCTU to enable remote data entry at sites where this is feasible. This database will provide built-in data-validation checks with quality control (QC) checks performed by checking a predefined percentage of CRF data against data entered into the database. In addition, on-site monitoring will enable source document verification (SDV) of records.

Patient-reported outcome measures (PROMs), including questionnaires and diaries, may be collected directly to the eCRF using a secure and controlled REDCap database. An automated email reminder will be sent to participants to remind them to complete the questionnaires and diaries every 12 weeks. Alternatively, participants can complete paper questionnaires and diaries to be entered by the central study team.

All patient-identifiable data, such as Consent Forms, screening and identification logs will be stored in the investigator site files in secure locked cabinets and/or offices, accessible only to delegated members of the study team. Secure methods of data transfer will be used to return CRFs to the coordinating site for centralised data entry, monitoring, QC in compliance with GCP. A copy of the CRF will be held at the site in accordance with GCP.

### Follow-up procedures

The study duration allows for follow-up to a maximum of 12 months with data collection at 3, 6 and 12 months post initiation of therapy. Primary outcome data will be collected at 3 months. Each participant will have a minimum of 3, 6 and 12 months’ follow-up data for collecting the primary and secondary outcomes. In addition, PAC-SYM, PAC-QOL and EQ-5D-5 L and EQ-VAS will be recorded at the 1-month visit; this is to capture information on early nonresponders and to better understand and characterise this group of patients. Participants will leave the study and return to ‘routine clinical care’ as determined within their local NHS institution (or be recruited to subsequent trials). Alternatively, they may wish to proceed to enrollment in the next WP (study 3 – Laparoscopic Ventral Mesh Rectopexy) within the CapaCiTY programme.

### The following data will be collected at each visit up to 12 months

- Validated symptom and QOL questionnaires (PAC-SYM and PAC-QOL). Validated generic QOL questionnaires: EQ-5D-5 L descriptive system and EQ-VAS. Note: EQ-VAS has a standard deviation (SD) of approximately 30 points: a 10% difference in VAS deemed clinically significant can be detected with the large sample sizes proposed
- Patient Health Questionnaire-9 (PHQ-9) [53–55]
- Generalised Anxiety Disorder Questionnaire (GAD-7) [56]
- Depression, anxiety and somatisation modules of the Patient Health Questionnaire [53–56] and the Illness Perception Questionnaire [57]
- Global patient satisfaction/improvement score (VAS) and whether they would recommend each treatment experienced to other patients
- Potentially modifiable cognitive and behavioural psychological variables shown to predict onset and perpetuation of other functional bowel symptoms: negative perfectionism, avoidant and ‘all or nothing’ behaviour subscales of the Behavioural Response to illness Questionnaire (CC-BRQ), and the Brief Illness Perception Questionnaire BIPQ (CC)
- A 2-week Patient Diary (for 2 weeks prior to each assessment at 3, 6 and 12 months) to record bowel frequency and whether each evacuation was spontaneous (no use of laxatives) and/or complete; the patient journal will also capture concurrent medication, health contacts, and time away from normal activities (including work). Patients will be contacted by telephone to remind them to start the diary. If a patients forget to do this, then it is acceptable for them to start recording the diary on the day that they are seen in clinic and for this to be collected 2 weeks later
- Resource use data (using patient journals as a prompt and including concomitant medication use)
- Irrigation Diary to record frequency and volume of irrigation and any AEs

### Laboratory assessments

Serum or urine pregnancy testing may be performed as per standard care for any women of equivocal status undergoing radiological assessments (INVEST).

## Radiology assessments

The whole gut transit study usually (90% patients) involves the use of a single, plain abdominal radiograph (in 10% patients, a maximum of two may be required to image the whole abdomen and pelvis). This procedure forms part of routine clinical care for patients with CC at many NHS centres. All practitioners (radiologists, radiographers, etc.) directing these studies will hold appropriate IR(ME)R certification.

### Participant withdrawal (including data collection/retention for withdrawn participants)

Individual participants will be able to withdraw from treatment at any time by notifying health care professionals involved with the study, and return to routine care without prejudice. Data will be retained for analysis from all participants after the point of consent and recruitment.

Criteria for withdrawal from treatment:

#### Participant develops any of the following exclusion criteria

- Participant becomes pregnant or intends to become pregnant (only in baseline and intervention phases)
- Participant is subsequently diagnosed with a proven cause for secondary constipation, e.g. Parkinson’s disease or bowel obstruction
- Participant requires new medication with proven effects on bowel function, e.g. opioids
- Participant develops significant intercurrent illness precluding participation
- Participant requires surgery or other intervention (other than minor ops) during treatment or follow-up phase
- Participant develops acute psychological problem causing safety concern
- Adverse events secondary to therapy (bleeding, anal fissure, ulceration, pain, bowel perforation) – relative indications for withdrawal depending on the views of the patient and physician. (Note: bowel perforation is an absolute indication for withdrawal)
- Elective withdrawal

#### Loss to follow-up (no further interventions or follow-up data collected)

- During follow-up (up to 12 months), participants may be withdrawn from the trial if they become lost to follow-up (LTFU) after at least three failed attempts by research staff to make contact via two different methods (e.g. telephone and letter)
- Participant chooses to withdraw and does not wish to participate in follow-up data collection
- Death or significant incapacity making follow-up data collection impossible

### End of study definition

The end of study is defined as the last patient last visit. The sponsor, REC and local R&D departments will be informed of end of study and site closure and archiving procedures initiated.

### Criteria for early termination

If the Data Monitoring and Ethics Committee (DMEC), Programme Steering Committee (PSC), Research Ethics Committee (REC) or sponsor determine that it is within the best interests of the participants or trial to terminate the study, written notification will be given to the chief investigator (CI). This may be due to, but not limited to: serious safety concerns, serious breaches, acts of fraud, critical findings or persistent noncompliance that negatively affects patient safety or data integrity. If the study is terminated participants will be returned to the NHS normal follow-up and routine care.

### Qualitative interviews

The purpose of this qualitative enquiry is to complement the quantitative study of TAI. A phenomenological methodology will be employed and qualitative data will be collected in parallel with the quantitative study. Participants will be recruited separately from the quantitative study, with separate PISs and consent processes.

### Sampling

A purposive sample of approximately 35 patients will be invited to interview upon completion of irrigation training and then again at 6 months. Participants do not have to participate in both sets of interviews; a separate set of patients can be interviewed at 6 months. Recruitment can be extended if data saturation is not accomplished by the 35th patient. Data saturation is defined as the point at which no new or relevant themes emerge. Inclusion and exclusion criteria are as above. Participants will be selected from a sampling grid of potential interviewees to reflect a range of ages, geographical locations and, where possible, other pertinent attributes such as ethnicity and gender. An approximately equal number of patients will be selected from each trial arm as follows:

- Seventeen patients undergoing low-volume anal irrigation and 18 patients undergoing high-volume irrigation and including those who discontinue early (before 3 months), later (3–5 months), those who continue with their allocated treatment, and those who switch
- In addition, approximately 10 health professionals involved in delivering the treatment will be interviewed. These health care professionals will be evenly distributed across participating centres

### Data collection

All participants will be told that they might be invited for interview when they are initially informed about the study. Participants will be contacted by a member of the clinical team and if interested in being interviewed a separate PIS will be provided. Participants will be offered a semistructured interview in a clinic room or in their own home according to their preference, and will be offered a chaperone to be present if they would prefer. Professionals will be interviewed in a clinic setting. Following written consent, the interviews will be recorded on a digital dictaphone and transcribed into a pseudonymised (alphanumeric code) text document. Interviews will be conducted by an experienced qualitative researcher working within the wider CapaCiTY research programme. A clinical research fellow at UHND and/or a health research methodologist at Durham University will conduct interviews recruited from the Durham site.

Interviews will explore health professionals’ and participants’ experiences of recruitment, individual interventions, their training and delivery, and patients’ views about outcome measures. A topic guide for each of the interviews and focus groups, informed by the existing literature and our patient advisors, will be developed.

### Timing

Patients will be invited to one-to-one interviews on completion of training and will be interviewed a maximum of 4 weeks after training to maximise recall. Patients will be recalled up to 6 months after training and offered an interview. The patients interviewed at baseline do not have to be the same as those interviewed up to 6 months. Interviews will be conducted throughout to capture relatively early and later experiences and perceptions of the interventions.

### Analysis

Interviews will be digitally recorded, anonymised, transcribed verbatim and analysed using a thematic analysis and NVivo8 software (QSR International Ltd., Warrington, UK) for data management. Data analysis will be developed as outlined by Fereday and Muir-Cochrane [58] in the first instance by mapping key concepts derived from the transcripts (‘charting’) and extracting emergent themes from the transcripts. Professor Norton will coordinate and conduct analysis, while for the purposes of Christopher Emmett’s MD, independent analysis will be conducted by CE and Dr. Helen Close. Emergent themes, together with captured observational data, will form the basis of analytical interpretation. Data will be handled in a confidential manner at all times, and only transferred on encrypted media or via secure electronic transfer.

## Statistical considerations

### Sample size

PAC-QOL is a 28-item disease-specific measure, with each item scored 0–4, and providing an aggregate score 0– [34]. Superiority of either low-volume or high-volume anal irrigation is demonstrated by a 10% scale difference (or more), or 0.4, with a variance estimate conservatively set at SD = 1 from the published medical literature [59]. To detect an effect size of 0.4 (mean/SD = 0.4) between the two groups with 90% power and 5% significance at 3 months requires 133 patients per arm, and 266 total. Allowing for an anticipated 10% loss to follow-up (LTFU), then 300 patients will be recruited.

### Clinical outcomes

A full analysis plan will be signed off before allocation codes are made available to the statistician. The codes will not indicate which treatment arm is which so that as far as possible the statistician will remain blind to allocation throughout the analysis. All analyses will be by the intention-to-treat principle. The primary outcome will be PAC-QOL as a continuous variable, analysed at 3 months while the quarantine period is in effect. The proportion of patients continuing with the initial therapy system will be recorded, and the PAC-QOL scores will be analysed using a linear mixed model with a random effect for centre and fixed effects for intervention, trial stratification variables (participants are stratified by sex and women by centre) and baseline PAC-QOL. Secondary outcomes will be analysed using the principles outlined above for the primary outcome.

Exploratory modelling will be conducted for baseline characteristics: measures of chronic pain, autonomic, joint hypermobility, cognitive, behavioural and mood variables share a common hypothesis that they are detrimental to the success of all treatments, i.e. they perpetuate illness in spite of therapy. We will investigate a maximum of three interactions between treatment and baseline characteristics. These will be described in the SAP a priori. Appropriate regression models, including interaction terms, will be developed to determine the influence of these pretreatment characteristics on the success of treatments in all WPs.

Life table data for any irrigation will be presented by initial therapy and for specific therapy from date of commencement. Survival analysis will be presented using Kaplan-Maier analysis and adjusted using Cox regression. Exploratory analysis will be considered to identify characteristics of subgroups with greatest persistent benefit from irrigation. These will be described in the SAP a priori.

Analysis will be performed using proprietary software, (Stata Corp., College Station, TX, USA). *P* < 0.05 will be taken to indicate statistical significance. No analyses will be conducted until an analysis plan has been written, reviewed by an independent statistician and signed off.

Multiple imputation will be considered to address missing covariate values. Details of any imputation to be performed will be described in the SAP which will be finalised after initial checks on completeness of the data but before performing any analysis or unblinding of the data.

### Health economic outcomes

The patient journal will facilitate the capture of health economic data which will be recorded on the CRF at each visit. This will be combined with the initial cost of the device and weekly consumables.

Within-trial stochastic analysis will compare the cost/success and cost/quality-adjusted life year (QALY) of anal irrigation. Patient-level cost-effectiveness analysis will use standard bootstrapping methods to generate cost-effectiveness acceptability curves exploring value for money. Within-cohort combined stochastic/probabilistic epidemiological models will be used to assess irrigation and surgery options, exploring relative effectiveness and cost-effectiveness according to patient characteristics.

Cost-effectiveness models that extrapolate beyond 3–6 months’ duration are problematic in adult constipation, as subsequent care and outcomes are contingent upon subsequent care received and the underlying disease process. However, the programme of WPs, and inclusion of time to failure data capture, provides a unique opportunity to construct probabilistic models exploring optimal pathways from effectiveness and cost-effectiveness perspectives.

Since patients will (within the CapaCiTY programme) be followed along a pathway that includes a series of steps of care, it will be possible to construct costs and outcomes for a range of patient pathways providing comparative longer-term cost-effectiveness estimates. Patient-level data from recruitment through the various WPs will be used to construct pragmatic, probabilistic models to explore optimal pathways from effectiveness and cost-effectiveness perspectives.

Analyses from NHS and societal perspectives will be supported by recording relevant resource use during each WP, and a common panel of outcomes. Adjustment for time preference will be at the socially accepted rate for cost-effectiveness analyses (currently 3.5%/annum for costs and benefits).

### Data analysis for MD thesis

The study will form the basis of a thesis for an MD at Durham University by a research fellow (Christopher Emmett) at University Hospital of North Durham (UHND). Patients recruited at UHND and the Royal Victoria Infirmary, Newcastle upon Tyne up to 1 October 2016 (estimated 50 patients) will be analysed in this thesis, including those recruited to the qualitative arm of the study at this site. These patients will have a minimum of 3 months of study data. The release of data from the UHND and Newcastle sites for this purpose has been approved by the chief investigator (CI) on the condition that it may be used for thesis examination but is not published or made publically available until the CapaCiTY programme results are published in full. The qualitative data from the Durham site may be published separately as agreed.

## Laboratories (if applicable)

Serum pregnancy testing will be performed by local NHS biochemistry laboratories.

## Products, devices, techniques and tools

### Devices

There are no investigative medicinal products or investigative devices under study. The following is a list of all devices routinely used in clinical care and none are specific to the research itself. All are CE-marked and approved for use in the UK.

1. Disposable proctoscope (supplier as local NHS practice). This will be commonly be used as part of clinical examination at baseline and is also used to introduce balloon catheters into the rectum during INVEST
2. High-resolution anorectal manometry (HRAM system + Unisensor HRAM catheter (200 uses) and balloons, software, cables, calibration kit, isolation transformer and laptop. Insertion and use are outlined under the ‘Interventions’ section (equipment provided at study outset)
3. Standard anorectal manometry catheter, balloons, software, cables, calibration kit and associated equipment; standard equipment in many NHS centres for performing anorectal physiology. Can be used as an alternative where high-resolution manometry is not available (part of INVEST – see above)
4. Balloon catheters for balloon expulsion test (part of INVEST – see above)
5. Radio-opaque markers for colonic transit study: various suppliers (part of INVEST – see above)
6. Standard departmental X-ray equipment (part of INVEST – see above)
7. Peristeen™ anal irrigation system (Coloplast), Qufora® Balloon™/Qufora Toilet anal irrigation systems (MBH-International): established anal irrigation systems available on prescription in NHS practice. Other systems with the same mechanism of action may also be used (dependent on local funding and prescribing arrangements)
8. Qufora® Mini anal irrigation system (MBH-International): established anal irrigation system available on prescription in NHS practice

All devices are maintained, calibrated and serviced according to standard NHS policies and procedures according to manufacturer’s guidance. Training on devices is provided by the supplier’s representatives. Additional study SOPs and training will be provided to ensure standardisation across sites, but will be in line with current NHS standard practice.

### Data collection tools

The permissions/licenses to use the below instruments have been sought on the understanding that sites are permitted to utilise these within this study only, they will be provided to sites as part of the CRF for the study:

- PAC-QOL score: from MAPI Research Trust
- PAC-SYM score: from MAPI Research Trust
- EQ-5D-5 L: from EuroQol

The below-listed questionnaire-based tools are free to use within the public domain and will be provided to sites as part of the CRFs for the study.

- Depression, anxiety and somatisation modules of the Patient Health Questionnaire
- Illness Perception Questionnaire
- Composite Rome III/Cleveland Clinic Constipation Questionnaire: free to use
- Brief, chronic pain, autonomic and joint hypermobility: free to use
- Negative perfectionism
- Avoidant and ‘all or nothing’ behaviour subscales of the Behavioural Response to Illness Questionnaire

## Safety reporting

### Adverse events (AEs)

An AE is any untoward medical occurrence in a subject to whom an intervention has been administered, including occurrences which are not necessarily caused by, or related to, that intervention. An AE can, therefore, be any unfavourable and unintended sign (including an abnormal laboratory finding), symptom or disease temporarily associated with study activities.

#### Notification and reporting adverse events or reactions

The anal irrigation systems are in widespread and established clinical use throughout the NHS with known AEs occurring (22%) being mostly low grade and reversible. All trial interventions are as per the standard care provided within the NHS for CC. Related AEs will be recorded on the CRF. Serious adverse events (SAEs) will be recorded on the CRF and in the medical notes to enable assessment and reporting in line with sponsor and regulatory requirements. Causality will be at the discretion of the health care provider (e.g. research nurse, physiotherapist, PI or delegated member of team). These will be assessed as outlined below.

Trial participants will be advised to seek medical support from their GP for any unrelated signs, symptoms or disease or aggravation of underlying symptoms.

### Serious adverse event (SAE)

In other research other than CTIMPs, a SAE is defined as an untoward occurrence that:

1. Results in death
2. Is life-threatening.
3. Requires hospitalisation or prolongation of existing hospitalisation
4. Results in persistent or significant disability or incapacity
5. Consists of a congenital anomaly or birth defect, or
6. Is otherwise considered medically significant by the investigator

An SAE occurring to a research participant should be reported to the sponsor and Main Research Ethics Committee (MREC) where, in the opinion of the CI, the event was:

- Related – that is, it resulted from administration of any of the research procedures, and
- Unexpected – that is, the type of event is not listed in the protocol as an expected occurrence (see Additional file 3)

### Notification and reporting of SAEs

Serious adverse events (SAEs) that are considered to be ‘related’ and ‘unexpected’ are to be reported to the sponsor within 24 h of learning of the event and to the MREC within 15 days in line with the required timeframe. For further guidance on this matter, please refer to the HRA website and Joint Research Management Office (JRMO) SOPs.

### Expected SAEs

The following SAEs are expected to occur rarely in this patient population and will not be reported:

- Hospital admission for exacerbation of constipation symptoms including impaction
- Hospital admission for unrelated elective surgical procedures or accidental injury

### Urgent safety measures

The CI may take urgent safety measures to ensure the safety and protection of the clinical trial subjects from any immediate hazard to their health and safety. The measures should be taken immediately. In this instance, the approval of the REC prior to implementing these safety measures is not required. However, it is the responsibility of the CI to inform the sponsor and the MREC (via telephone) of this event immediately.

The CI has an obligation to inform both the MREC in writing within 3 days, in the form of a substantial amendment. The sponsor, JRMO, must be sent a copy of the correspondence with regards to this matter. For further guidance on this matter, please refer to the HRA website and JRMO SOPs.

### Annual safety reporting

The CI will send the Annual Progress Report to the MREC using the HRA template (the anniversary date is the date on the MREC ‘favourable opinion’ letter from the MREC) and to the sponsor. Please see the HRA website and JRMO SOP for further information.

### Overview of the safety reporting responsibilities

The CI/PI has the overall responsibility for oversight of safety reporting. The CI/PI also has a duty to ensure that safety monitoring and reporting is conducted in accordance with the sponsor’s requirements.

## Monitoring and auditing

The PCTU quality assurance (QA) manager will conduct a study risk assessment in collaboration with the CI. Based on the risk assessment, an appropriate study monitoring and auditing plan will be produced according to PCTU SOPs. This monitoring plan will be authorised by the sponsor before implementation. Any changes to the monitoring plan must be agreed by the PCTU QA manager and the sponsor.

Audit definition:

‘A systematic and independent examination of trial-related activities and documents to determine whether the evaluated trial-related activities we reconducted, and the data were recorded, analysed and accurately reported according to the protocol, sponsor’s SOPs, Good Clinical Practice (GCP) and the applicable regulatory requirement(s).’

A study may be identified for audit by any method listed below:

1. A project may be identified via the risk assessment process.
2. An individual investigator or department may request an audit.
3. A project may be identified via an allegation of research misconduct or fraud or a suspected breach of regulations
4. Projects may be selected at random. The Department of Health states that trusts should be auditing a minimum of 10% of all research projects
5. Projects may be randomly selected for audit by an external organisation

Internal audits may be conducted by a sponsor’s or a funder’s representative according to JRMO/NIHR SOPs.

## Safety considerations

Patients recruited who have not had previous INVEST procedures conducted within the last 12 months will undergo a radiological procedure (whole gut transit) using ionising radiation as outlined above. The average dose of this procedure (approximately 0.1 mSv) is equivalent to about 2.5 weeks’ annual background radiation dose from living in the UK Further, these investigations would be carried out in routine clinical practice in many centres for patients at the same point as recruitment to this study.

Regarding the intervention, anal irrigation is associated with a very low incidence of bowel perforation, as well as other side effects (bleeding, pain, ulceration, painful haemorrhoids, anal fissure). Patients will be counselled regarding these risks as part of the process of informed consent. In addition, they will be trained in the correct use of the device prior to commencing therapy. All related AEs and all SAEs will be recorded and therapy suspended while these are investigated.

## Trial committees

The project will be under the auspices of the CI and the PCTU. The project will be overseen by a Programme Steering Committee (PSC).

The composition and responsibilities of the PSC will comply with the NIHR guidance and PCTU SOP on Trial Oversight Committees. The role of the PSC is to provide overall supervision of the study on behalf of the sponsor and funder to ensure that study is conducted in accordance with the principles of Good Clinical Practice (GCP) relevant regulations.

The responsibilities of the PSC will include:

- Ensuring that the views of users and carers are taken into consideration
- Advising on the trial protocol
- Advising on changes in the protocol based on considerations of feasibility and practicability
- Assisting in resolving problems brought to it by the Programme Management Group (PMG)
- Monitoring the progress of the trial and adherence to protocol and milestones
- Considering new information of relevance from other sources
- Considering and acting on the recommendations of the Data Monitoring Committee (DMC), sponsor and/or MREC
- Review initial reports and papers for publication

The PSC will meet to review the protocol before the start of the programme and then soon after the first participants are recruited and either meet or teleconference every 6 months thereafter throughout the lifetime of the programme.

Representatives of the trial sponsor and the funder will be invited to attend.

A PMG made up of core staff from the coordinating centres and the PCTU will meet monthly initially during study set-up and then less frequently, every 2 months. The PMG will be responsible for day-to-day project delivery across participating centres, and will report to the PSC.

An independent Data Monitoring and Ethics Committee (DMEC) will be convened. The DMEC will meet at least 4 weeks prior to the PSC to enable recommendations to be fed forward.

A DAMOCLES charter will be adopted, and the project team will provide the DMEC with a comprehensive report, the content of which should be agreed in advance by the chair of the DMEC and follow guidelines set out in the charter.

A Constipation Research Advisory Group (CRAG) will be formed as part of a well-developed Patient and Public Involvement (PPI) strategy at QMUL. This advisory group will comprise eight patients and two lay members derived from London and Durham. This group will have geographical diversity (north and south) and a disease-appropriate demographic (eight women, two men). The CRAG will be involved in:

- Review of PISs, booklets, diaries and advertising/marketing materials
- Project management by representation on the PSC
- Parallel qualitative analysis
- Dissemination of results and lay summaries
- Presentations at local research events
- Patient focus groups and workshops

## Project management

### Local coordination

Each participating centre will identify a site-specific PI who will nominate a local contact for that centre (this may be themselves). The PI and local contact will:

- Be familiar with the trial
- Liaise with the PCTU and the PMG
- Ensure that all staff involved in the trial are informed about the trial and have received requisite training
- Ensure that mechanisms for recruitment of eligible participants, including the availability of participant information and data collection tools, are in place; monitor their effectiveness and discuss the reasons for nonrecruitment with relevant staff
- Ensure that site staff collect necessary trial data and perform quality checks
- Notify the CI of any SAE’s
- Make data available for verification, audit and inspection processes as necessary, and respond to requests for documentation and data required for centralised monitoring
- Ensure that the confidentiality of all information about trial participants is respected by all persons

### Site initiation and training

A central study launch meeting and/or site initiation will be conducted with each site. This will include training in the trial protocol and SOPs, such as data collection, randomisation and taking informed consent. Evidence of appropriate training, local approvals and essential documentation will be required before participants being enrolled at each site. Training will be documented on training logs.

### Project timetable, milestones and projected recruitment

The PMG will be responsible for monitoring adherence to the study timelines and expected recruitment rates. Regular reports will be produced to enable deviations from the project plan to be identified and contingencies planned, discussed and executed in a timely fashion.

Projected recruitment dates are:

- 1 Aug 2015: first participant
- 31 Apr 2016: 100 participants
- 30 Nov 2016: 200 participants
- 30 Jun 2017: 300 participants
- 30 Oct 2017: last patient intervention
- 31 Apr 2018: 3-month primary endpoint
- 31 Oct 2018: 12-month secondary endpoint

## Discussion

The CapaCiTY 02 study is a large and potentially very rich study in terms of hypothesis-testing and generating robust evidence. As previously noted, its primary aim of establishing superiority of one system of irrigation over another will provide valuable information that can be used to guide the choice of therapy in patients with CC. Additionally, the study aims to explore health economic outcomes, and will also evaluate the association between pretreatment baseline characteristics (e.g. psychological profile, joint hypermobility, colonic transit, anorectal physiology) and treatment success. Alongside these elements, a qualitative component of the study will explore the lived experiences of patients and health care professionals who are using irrigation, or training patients in its use.

The multisite nature of the study, along with the broad range of outcome measures being employed, could potentially lead to several practical challenges in implementing the study protocol. Attempts have been made to anticipate and address these before commencing study recruitment. Prestudy site feasibility questionnaires were circulated to all sites wishing to participate, and these were used to identify the key components of irrigation training and treatment at each site. The training process described in this protocol aims to be as applicable as possible to as broad a range of sites as feasible, without causing the study sites to make significant alterations to their standard practice.

The protocol also aims to be flexible as regards pretreatment investigations. These have been limited to anorectal physiology and a transit study (see section above: ‘INVEST’). It was felt that these provided important information necessary for characterising patients before starting treatment, thereby allowing analysis of the relationship between pretreatment characteristics and treatment success. It was decided, as few sites had access to HRAM, that standard manometry was sufficient for the purposes of this study. This increased participation by allowing sites not in possession of the necessary high-resolution equipment to still recruit to the study.

It is recognised that the study design has several limitations. From a methodological perspective, the fact that neither participant nor assessor blinding was feasible (due to the nature of the treatment and the nature of the outcome data being collected), leads to the possibility of performance bias and reporting bias, as both participants and assessors will (consciously or unconsciously) have particular preconceived ideas about the likely efficacy of each system. Attempts have been made, from a methodological and operational perspective, to limit the impact of this. The fact that every patient receives treatment is important, as it is a reasonable assumption that the placebo effect for each system is similar, thereby meaning that any observed difference between systems is a genuine one. Additionally, the option of switching systems after 3 months is designed to allow participants who have not had success with their original system to try the other one. This means that patients do not spend too long on ineffective treatment, and also allows longer-term data (more than 3 months) to evaluate the effectiveness of the treatment as a whole in the long term.

As can be seen from the ‘Trial status’ section below, recruitment nationally has fallen below the planned rate of recruitment. Several reasons for low recruitment have been identified through discussions with participating sites; these are mainly the result of variation in local practice (making the protocol difficult to implement), as well as service pressures and the pressure on research teams from doing more than one study in the CapaCiTY programme. This highlights the difficulties in implementing multisite studies, and even though attempts were made before study commencement to ensure sufficient flexibility in the proposed study design, problems have nonetheless been encountered.

Since recruitment has opened, recruitment rates at each study site are monitored and monthly meetings are held to discuss progress and to identify problems at an early stage. Teleconferences have been held with recruiting sites in order to discuss and resolve barriers to recruitment.

## Trial status

As of 31 August 2016, the study has seven sites open to recruitment. The first patient was enrolled on 15 October 2015. Currently, 39 patients have been screened and 22 randomised. Of these, two have withdrawn (elective withdrawal – no reason given).

## Additional files

Additional file 1: CapaCiTY programme overview. (DOCX 152 kb)

Additional file 2: SPIRIT Checklist. (DOCX 66 kb)

Additional file 3: Communication organogram for reporting serious adverse events (SAEs). (DOCX 52 kb)

Additional file 4: Consent Form (quantitative study). (DOCX 64 kb)

Additional file 5: Consent Form (qualitative study). (DOCX 62 kb)

## Abbreviations

AE: Adverse event
BD: Twice per day
BIPQ: Brief Illness Perception Questionnaire
BNF: *British National Formulary*
CC: Chronic constipation
CI: Chief investigator
CPCPH: Centre for Primary Care and Public Health
CRF: Case Report Form
DMEC: Data Monitoring and Ethics Committee
EC: European Commission
EQ-5D: EuroQol Health Outcome measure
EQ-VAS: EuroQol Visual Analogue Scale
FDD: Functional defaecation disorder
GAD-7: Generalised Anxiety Disorder Questionnaire
GAfREC: Governance Arrangements for NHS Research Ethics Committees
HRA: Health Research Authority
HT: Habit training
HTBF: Habit training incorporating direct visual biofeedback
INVEST: Standard panel of radio-physiological tests of colonic and anorectal function
JRMO: Joint Research Management Office
LTF: Lost to follow-up
NDUHT: North Durham University Hospital Trust
NHS R&D: National Health Service Research & Development
NHS REC: National Health Service Research Ethics Committee
PAC-QOL: Patient Assessment of Constipation Quality of Life questionnaire
PAC-SYM: Patient Assessment of Constipation Symptoms Questionnaire
PCSG: Primary Care Society for Gastroenterology
PCTU: Pragmatic Clinical Trials Unit
PHQ-9: Patient Health Questionnaire − 9
PI: Principal investigator
PIS: Participant Information Sheet
PMG: Programme Management Group
PPIG: Patient and Public Involvement Group
PRO: Patient-reported outcome
PSC: Programme Steering Committee
QA: Quality assurance
QC: Quality control
QOL: Quality of life
RAIR: Recto-anal Inhibitory Reflex
RCT: Randomised controlled trial
REC: Research Ethics Committee
SAE: Serious adverse event
SAP: Statistical analysis plan
SD: Standard deviation
SDV: Source document verification
SOP: Standard operating procedure
TAI: Trans-anal irrigation
TDS: Three times per day
VAS: Visual Analogue Scale
WP: Work package signature page
