# Supplementary material for: Low-volume versus high-volume initiated trans-anal irrigation therapy in adults with chronic constipation: study protocol for a randomised controlled trial
Source: Trials. 2017 Mar 31;18:151. doi: 10.1186/s13063-017-1882-y (PMC5374566; doi:10.1186/s13063-017-1882-y)
Supplement: Supplementary file 4 — Consent Form (quantitative study). (DOCX 64 kb) [file 13063_2017_1882_MOESM4_ESM.docx]

**STUDY CONSENT FORM**

Title of Project: **Chronic Constipation Treatment Pathway, Study 02**

Name of Researcher: **Professor Yan Yiannakou, professor of Neurogastroenterology**

**County Durham and Darlington NHS Foundation Trust**

**(**[**Yan.yiannakou@nhs.net**](mailto:Yan.yiannakou@nhs.net)**. Tel: 07584387147)**

| **Study ID:** |  |  | **-** |  |  |  | **-** |  |  |  |  |
| --- | --- | --- | --- | --- | --- | --- | --- | --- | --- | --- | --- |

|  | **Place initials in each box** |
| --- | --- |
| 1. I confirm that I have read and understand the Patient Information Sheet dated **22 June 2015** (version 2) for the above study. I have had the opportunity to consider the information, ask questions and have had these answered satisfactorily. |  |
| 1. I understand that my participation is voluntary and that I am free to withdraw at any time without giving any reason, without my medical care or legal rights being affected. |  |
| 1. I understand that relevant sections of my medical notes and data collected during the study may be looked at by individuals from Queen Mary, University of London, from regulatory authorities or from the NHS Trust, where it is relevant to my taking part in this research. I give permission for these individuals to have access to my records. |  |
| 1. I agree to undergo GI Physiological tests including tests using X-rays. |  |
| 1. I agree for my contact details to be passed to study interviewers so I can be contacted about taking part in one to one interviews |  |
| 1. I agree to my GP being informed of my participation in the study. |  |
| 1. I agree to take part in the above study. |  |

|  |  |  |  |  |
| --- | --- | --- | --- | --- |
| *Print Name of Participant* |  | *Date* |  | *Participant’s Signature* |
|  |  |  |  |  |
| *Print Name of person taking consent* |  | *Date* |  | *Signature of person taking consent* |
