# Supplementary material for: Low-volume versus high-volume initiated trans-anal irrigation therapy in adults with chronic constipation: study protocol for a randomised controlled trial
Source: Trials. 2017 Mar 31;18:151. doi: 10.1186/s13063-017-1882-y (PMC5374566; doi:10.1186/s13063-017-1882-y)
Supplement: Supplementary file 5 — Consent Form (qualitative study). (DOCX 62 kb) [file 13063_2017_1882_MOESM5_ESM.docx]

**INTERVIEW CONSENT FORM**

Title of Project: **Chronic Constipation Treatment Pathway, Study 02**

Name of Researcher: **Professor Yan Yiannakou, Professor of Neurogastroenterology, County Durham and Darlington NHS Foundation Trust**

**(**[**Yan.yiannakou@nhs.net**](mailto:Yan.yiannakou@nhs.net)**. Tel:** **07584387147)**

| **Study ID:** |  |  | **-** |  |  |  | **-** |  |  |  |  |
| --- | --- | --- | --- | --- | --- | --- | --- | --- | --- | --- | --- |

|  | **Place initials in each box** |
| --- | --- |
| 1. I confirm that I have read and understand the Information Sheet dated **20 January 2015** (version **2**) for the above study. I have had the opportunity to consider the information, ask questions and have had these answered satisfactorily. |  |
| 1. I understand that my participation is voluntary and that I am free to withdraw at any time without giving any reason, without my medical care or legal rights being affected. |  |
| 1. I agree to take part in a 60 minute interview and I understand that this will be audio taped and transcribed. |  |
| 1. I agree to take part in the above study. |  |

|  |  |  |  |  |
| --- | --- | --- | --- | --- |
| *Print Name of Participant* |  | *Date* |  | *Participant’s Signature* |
|  |  |  |  |  |
| *Print Name of person taking consent* |  | *Date* |  | *Signature of person taking consent* |
