# Supplementary material for: Low-volume versus high-volume initiated trans-anal irrigation therapy in adults with chronic constipation: study protocol for a randomised controlled trial
Source: Trials. 2017 Mar 31;18:151. doi: 10.1186/s13063-017-1882-y (PMC5374566; doi:10.1186/s13063-017-1882-y)

Fig 1: DESIGN OVERVIEW
with approximate numbers at each stage

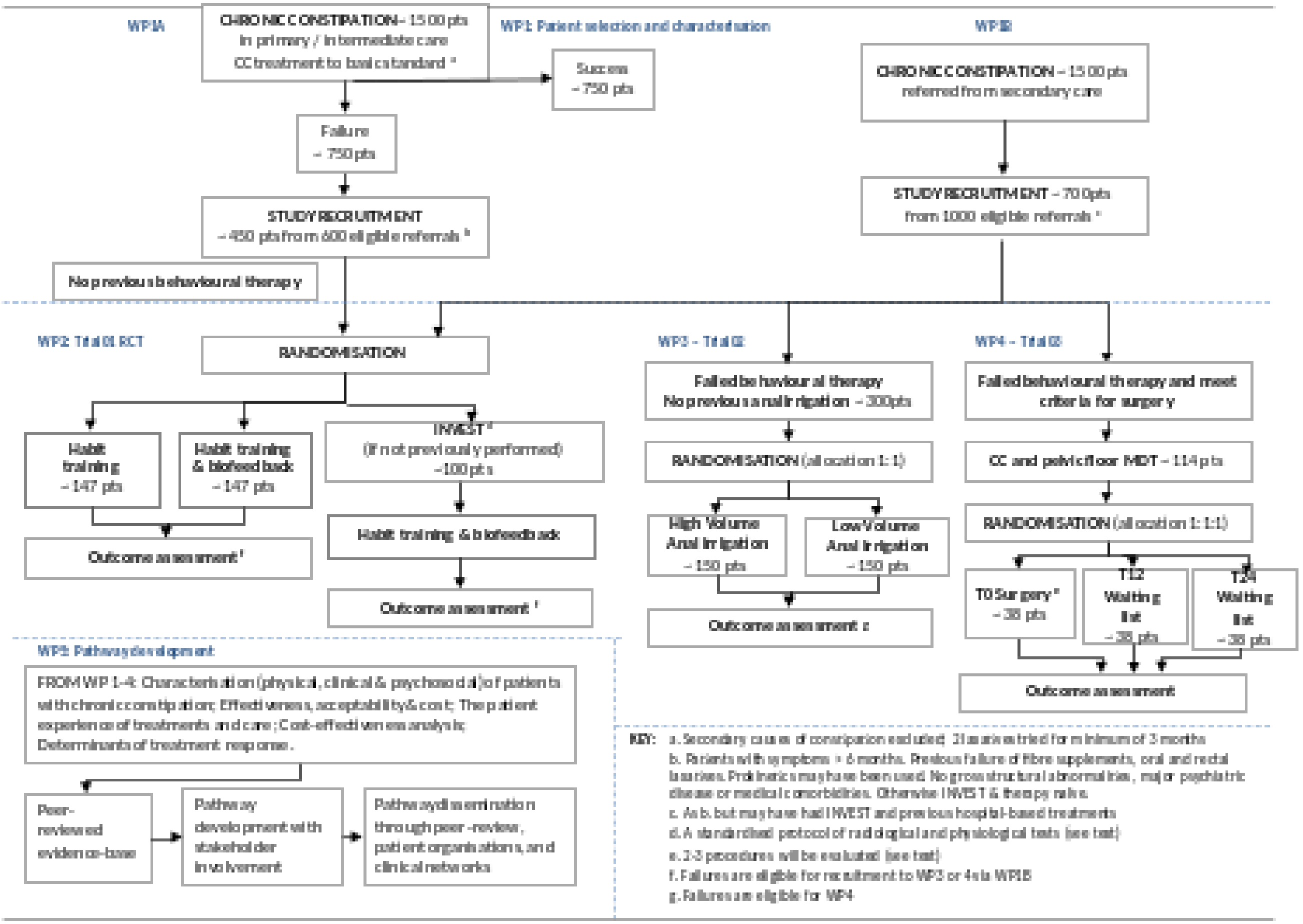

Supplement: Supplementary file 4 [file 13063_2017_1882_MOESM1_ESM.docx]
