# Supplementary material for: Low-volume versus high-volume initiated trans-anal irrigation therapy in adults with chronic constipation: study protocol for a randomised controlled trial
Source: Trials. 2017 Mar 31;18:151. doi: 10.1186/s13063-017-1882-y (PMC5374566; doi:10.1186/s13063-017-1882-y)
Supplement: Supplementary file 5 [file 13063_2017_1882_MOESM3_ESM.docx]

Figure 1: Communication organogram for reporting SAEs

SAE recorded on AE log and followed up until resolution

PI assesses SAE and reports to CI within **24 hours**, PI reports to local institution as per local protocol

CI reports related and unexpected SAE's to PCTU QA manager and Sponsor within **24 hours of PI becoming aware of the event**

CI reports related and unexpected SAE's to MREC within **15 days**

CI reports to DMC every **6-12 months**

CI reports **annually** to MREC

SAEs will be followed up until resolution.
